# On Chronic Pharyngitis

**Published:** 1892-09-17

**Authors:** 


### The Practitioner's Mirror and Retrospect.

[*The Editor will be glad to receive offers of co-operation and contributions from members of the profession. All letters should be addressed to* The Editor, The Lodge, Porchester Square, London, W.]

### MEDICINE.

### ON CHRONIC PHARYNGITIS.

That chronic pharyngitis not infrequently follows acute catarrhal pharyngitis which itself may be due to cold is sufficiently well known. The constant and generally improper use of the voice also is a frequent cause of the condition called "clergymen's sore throat." But there are many general pathological conditions which cause chronic pharyngitis very much more frequently than has hitherto been recognised, and there can be little doubt that it is largely due to this imperfect recognition of the real causes of the affection that so little success has followed our present methods of treatment.

Dr. Watson Williams, in a paper "On the Dyspeptic Sore Throat," read at the recent meeting of the British Medical Association, has directed attention to these general conditions. He referred to a form of sore throat, which is sometimes accompanied by very considerable suffering, and always by discomfort, exemplified by the case of a lady about fifty, who had suffered from a relaxed and painful throat for a year or two, with more or less constant hawking of pellets of mucus. She complained of increasing discomfort in the throat which had recently amounted at times to acute pain. The pain was referred to the back of the mouth, the pharynx, and to the hyoid region, and darting up to the ears. Latterly the pain had increased to such a degree that at times she was almost unable to swallow. At the commencement of a meal she was in pain, the odynphagia passed off in the course of the meal, and for a time she was nearly free from discomfort. She was very dyspeptic, complaining of heartburn and flatulence coming on an hour or two after meals.

On examination he found all the physical conditions usually associated with a moderate degree of chronic pharyngitis, but further, a brightly injected and fairly limited congestion of the fauces and soft palate. The bowels were constipated. She found the greatest relief from ordinary compound rhubarb pills, taken with her chief meals. One thing was certain, that by stimulating her liver with rhubarb she had obtained far more benefit than from all the local and general remedies that had previously been adopted.

The author had seen many similar cases in which the pain in the throat was sufficient to mask all other symptoms in the eyes of the patients, and has become increasingly convinced that a considerable proportion of cases with symptoms of subacute or chronic pharyngitis owe their origin to deficient hepatic action.

The question then arises as to how a torpid liver may give rise to acute pain in the throat as well as to chronic pharyngitis, for it is obvious that the majority of patients who suffer from hepatic congestion or obstruction, or from constipation and dyspepsia, do not complain of great pain in the throat.

It appears that some light may be thrown on the subject by the somewhat analogous condition of gouty sore throat to which Sir Morell Mackenzie drew attention in 1889. He referred to four typical cases that had come under his notice. One was an acute œdema of the uvula, disappearing upon sudden development of an ordinary attack of podagra; the second, a chronic inflammation of the posterior pillar of the fauces; the third, a gouty deposit around the crico-arytenoid joints on both sides, causing permanent dysphonia; and the fourth a gouty inflammation producing fungous ulcerations of the left ventricular band resembling cancer. The second of these cases was cured by colchicum, but the fourth case was cured by drinking the waters of Wiesbaden. Dr. Krishaber, of Paris, had diagnosed this case as cancerous, and Sir Morell candidly admitted that he himself was suspicious of its cancerous nature.

Dr. Harrison Allen has also made independent observations on the so-called gouty-sore-throat, referring to cases which "taken in connexion with the history and general condition, together with the results obtained by measures directed to the lithæmic state, indicate their dependance upon the so-called defective laboratory of the liver, concerning which but little is known." But he allows that he applies the term lithæmia with considerable lattitude and based upon clinical manifestations solely. Dr. Williams included many of the cases thus styled lithæmic as forms of the dyspeptic sore throat, for though, undoubtedly, they may occur in gouty subjects, the throat manifestations are the symptoms of a condition which is as frequently observed in those who are not by any means gouty, unless such a term be used in such a loose and general sense as to deprive it of all clinical value. Just as gout may possibly be the result of imperfect hepatic activity, so may other clinical conditions such as the throat condition to which he had alluded, of which the case referred to at the outset of these remarks is a very fairly typical example. A good illustration of the purely hepatic form of sore throat was furnished by the case of a medical man who came complaining very much of pain in the throat and soreness on swallowing, which had lasted about a fortnight, and referred more especially to the hyoid region. On examination, there was very little to account for all the discomfort; the tongue was slightly coated, and the fauces and soft palate were relaxed and vividly injected in patches. He was a little surprised at being questioned on the point, but admitted that he had recently been troubled with dyspepsia and headaches, which he thought might be due to inactivity of his liver, although the bowels acted regularly. He was advised to take small doses of euonymin and podophyllin at intervals, and his throat was almost immediately relieved.

It is, of course, generally recognised that portal congestion and the associated dyspeptic troubles are a common cause of chronic pharyngitis. The portal congestion may arise from enfeeblement of the heart or valvular lesions, from cirrhosis of the liver, or from frequently-recurring hepatic congestion from any cause. In these cases the chronic pharyngeal congestion is largely due to mechanical obstruction to the portal circulation, which results in the formation of a varicose communication between some radicals of the portal system and the general systemic veins; as between the hæmorrhoidal and the hypogastric, between the vein of Sappey, from the left branch of the portal vein to the epigastric and internal mammary, and, thirdly, between the veins of the stomach and the œsophageal to the pharynx, giving rise to the tortuous dilated œsophageal varices to which Dr. Stacy Wilson has recently redirected attention. Such mechanically-produced diffuse pharyngeal congestion undoubtedly is frequently attended with all the symptoms of chronic pharyngitis. But the intensely vivid and patchy injection of the painful throat to which attention is especially directed, is produced in a very different manner.

In typical cases the local appearances are fairly distinctive, for, instead of the dusky diffuse congestion of mechanical origin, or the diffuse congestion of pharyngitis arising from acute or sub-acute catarrhal attacks, we find an intensely vivid, well-demarcated patchy condition principally of the soft palate of the anterior and posterior pillars of the fauces and of the epiglottis. The pain is sometimes intense, and bearing no proportion to the often slight departure from the normal appearance of the parts involved. The frequent recurrence of this injection resulted in a chronic congestion indistinguishable in appearance from other forms of chronic pharyngitis.

